# Acute Kidney Injury and Atypical Features during Pediatric Poststreptococcal Glomerulonephritis

**DOI:** 10.1155/2016/5163065

**Published:** 2016-08-23

**Authors:** Rose M. Ayoob, Andrew L. Schwaderer

**Affiliations:** Division of Nephrology, Department of Pediatrics, Nationwide Children's Hospital, Columbus, OH, USA

## Abstract

The most common acute glomerulonephritis in children is poststreptococcal glomerulonephritis (PSGN) usually occurring between 3 and 12 years old. Hypertension and gross hematuria are common presenting symptoms. Most PSGN patients do not experience complications, but rapidly progressive glomerulonephritis and hypertensive encephalopathy have been reported. This paper reports 17 patients seen in 1 year for PSGN including 4 with atypical PSGN, at a pediatric tertiary care center. Seventeen children (11 males), mean age of 8 years, were analyzed. Ninety-four percent had elevated serum BUN levels and decreased GFR. Four of the hospitalized patients had complex presentations that included AKI along with positive ANA or ANCAs. Three patients required renal replacement therapy and two were thrombocytopenic. PSGN usually does not occur as a severe nephritis. Over the 12-month study period, 17 cases associated with low serum albumin in 53%, acute kidney injury in 94%, and thrombocytopenia in 18% were treated. The presentation of PSGN may be severe and in a small subset have associations similar to SLE nephritis findings including AKI, positive ANA, and hematological anomalies.

## 1. Introduction

The most common form of acute glomerulonephritis in children is poststreptococcal glomerulonephritis (PSGN) which usually occurs between 3 and 12 years old. PSGN occurs following infection with a nephritogenic strain of group A* Streptococcus* (GAS) in around 15% of cases [1]. PSGN is now recognized to be a immunologically mediated, nonsupporative complication of GAS [2]. Gross hematuria is present in 30–70% of patients while microscopic hematuria is present in all patients and hypertension occurs in 70% of patients requiring hospitalization [1]. Severe PSGN complications, hypertensive encephalopathy, and rapidly progressing glomerulonephritis occur in <10% and 1% of children hospitalized for PSGN, respectively [1]. The diagnosis is likely when hypocomplementemia is present during an acute glomerulonephritis and improves spontaneously within a few months [2]. Here we report a case series of severe PSGN cases seen at a pediatric tertiary care center over one year.

## 2. Methods

### 2.1. Study Type

One-year case series of children referred to a children's tertiary care hospital for PSGN.

### 2.2. Patients

After obtaining IRB approval (IRB09-00444), children seen with PSGN at Nationwide Children's Hospital (NCH) in Columbus, Ohio, between July 1, 2010, and June 30, 2011, were identified. Children between the ages of 3–18 years diagnosed with acute PSGN (ICD-9 code 580.0) seen in both inpatient and outpatient settings were identified. All patients included had hematuria based on urine dipstick, low serum complement (C3) that subsequently returned to normal within 3 months, and laboratory documentation of a streptococcal infection or household contact with a streptococcal infection. Patients with clinical or histological evidence of other underlying renal diseases were excluded.

### 2.3. Data Collection

Data obtained from medical records included age, sex, height, and weight as well as clinical course and complications. Labs recorded included complete blood counts, serum chemistries, and any additional serology or immunology collected at diagnosis or the following 4 months. For all patients, estimated glomerular filtration rate (eGFR) was calculated with the 2009 Schwartz formula [9].

### 2.4. Culture Isolates

Culture isolates were collected and transported to the Center for Disease Control and Prevention (CDC) Streptococcal Research Laboratory. The sample from* Patient 12* was collected from a throat culture and* Patient B* from fluid drained from a peritonsillar abscess. At the CDC, specimens were* emm* typed by sequencing polymerase chain reaction products [10]. Subtypes were assigned according to the CDC* emm* sequence database [11].

### 2.5. Renal Pathology

Percutaneous renal biopsies under ultrasound guidance were performed in 4 of the patients with unusual presentations or more severe disease. Specimens were fixed and embedded in paraffin using standard procedures and sections 1-2 *µ*m in thickness were stained with haematoxylin-eosin (HE), periodic acid-Schiff (PAS), Jones methenamine silver, and Masson's trichrome for pathologist examination under light microscopy. Immunofluorescence studies were performed for IgG, IgA, IgM, C1q, C3, and C4 antibodies and electron microscopy was performed on Epon embedded tissue stained with uranyl acetate and lead citrate.

### 2.6. Statistical Analysis

Descriptive statistics were reported as mean ± standard deviation for normally distributed data. Binomial variables were compared with a Fischer exact test if expected cell frequencies were less than 5; otherwise the Chi-square test was used. Continuous variables were evaluated by Student's *t*-test and considered significant for *P* < 0.05.

## 3. Results

### 3.1. Patient Demographics

After review of inpatient and outpatient records, 17 patients met inclusion criteria (Table 1). The mean age of included patients was 8 years and 65% (11/17) were male. Seventy-one percent (12/17) of the children presented between November and April and 88% (15/17) were Caucasian. All of the children examined had a current or preceding pharyngitis but none had a documented skin infection.

### 3.2. BP and Urine Lab Findings

Presentation and follow-up of BP and urine findings are presented in Table 2. A systolic and/or diastolic blood pressure (BP) ≥95th% tile for age, height, and sex at the time of presentation was noted in 88% (15/17) of patients and persisted into follow-up in 3 patients. All patients had hematuria.

### 3.3. Serum Lab Findings

Serum lab findings are presented in Table 3. All patients had C3 <80 mg/dL that normalized on follow-up. The mean nadir eGFR was 44.5 ± 22.9 mL/min/1.73 m^2^ that improved to 105 ± 19.4 on follow-up, although 18% (3/17) had eGFR that remained <80 on follow-up. Fifty-three percent (9/17) had serum albumin levels ≤3 g/dL. Additionally sixty-five percent (11/17) of the patients had hemoglobin levels <11 g/dL and 18% (3/17) presented with thrombocytopenia.

## 4. Atypical PSGN

Of the 13 patients hospitalized, 4 patients underwent renal biopsy due to concern for rapidly progressive glomerulonephritis. Additional serologic abnormalities were also found in these patients. Patients 3, 10, and 12 had positive antinuclear antibody (ANA) tests but negative* Crithidia* and each required renal replacement therapy (RRT) during their hospitalization (Table 4). Renal replacement therapy was performed on 3 patients and consisted of acute hemodialysis for the indications of fluid overload and/or uremia. The thrombocytopenia, when present, was not severe, with nadirs of 95 and 51 K/mm^3^ for patients 3 and 12, respectively. Patient 11 did not require RRT but did test positive for cytoplasmic anti-neutrophil cytoplasmic antibody (c-ANCA). All ANCA testing mentioned in this case series was performed by indirect immunofluorescence at the Ohio State Wexner Medical Center Laboratories. Patient 10 had new onset seizures of undetermined etiology. The glomeruli in all the biopsies showed diffuse proliferative glomerulonephritis with prominent infiltration of neutrophils and cellular crescents were found in 3 of the biopsies (Table 4). The throat culture collected from* Patient D *had GAS strain that was T nontypeable, M protein gene subtype 44.0 (*emm* 44.0).* Patient B*'s GAS strain from the fluid drained from a peritonsillar abscess was T type 2,* emm* 2.0.

### 4.1. Comparison to Past Studies

Comparison to past studies are presented in Table 5. In general our cohort appears to be more likely to have elevated BUN and lower GFR while hypertensive complications were similar.

## 5. Discussion

Our study was comparable to historical references with respect to presenting symptoms and mean age of presentation [2]. With respect to acute changes in renal function, our cohort noted higher BUN values and lower eGFR values. In the current study, 76% (13/17) of children had BUN >20 mg/dL and 94% (16/17) had eGFR <80 mL/min/1.73 m^2^ with the mean eGFR 45 mL/min/1.73 m^2^. Our patients also had more hypertensive complications.

A previous pediatric study of 153 children with PSGN reported that more than 50% of patients had hemoglobin ≤11 g/dL [12]. In our study, 52% (11/16) had levels at or below 11 g/dL. Significantly lower mean platelet levels were found compared to patients treated as outpatients in our study. Thrombocytopenia has only been associated with PSGN in a few case reports. The triad of hemolytic anemia, thrombocytopenia, and acute kidney injury occurred in a 13-year-old African American boy with a high ASO and low C3 [13]. A four-year-old Japanese boy was reported to have a low C3, “mild glomerulonephritis,” a negative throat culture and thrombocytopenia with platelet antigen-specific Fab fragment autoantibodies [14]. None of the patients in our series had elevated LDH or haptoglobin levels to suggest a hemolytic process. The anemia and/or thrombocytopenia created a diagnostic dilemma particularly when combined with a positive ANA titer or ANCA antibodies as the possibility of an underlying autoimmune or vasculitic process. Recent studies have indicated a higher rate of severe glomerulonephritis [15, 16]. Because the thrombocytopenia resolved around the same time as the nephritis, the patients with low platelets in our case series were not tested for thrombotic microangiopathies so we cannot rule out that underlying complement disorders may have been present. In the aforementioned publications from New Zealand, AKI was present in 67% of patients. Interestingly patients of Pacific Island and Maori descent were disproportionately affected and those of European descent were spared.

*Streptococcus pyogenes*, a beta-hemolytic bacterium of the Lancefield serogroup A, is also referred to as group A streptococci. Group A streptococci are usually typed based on their surface M proteins which function as virulence factors [17]. The common pharyngitis associated nephritogenic strains include M types 1, 4, and 25 and some M12 strains [2]. Over the last 40 years in the United States, an apparent decline in the incidence of PSGN is assumed to be due to the near eradication of streptococcal pyoderma due to improved hygiene and/or decreased prevalence of skin infection associated nephritogenic M serotypes [18]. Virtually no epidemiologic data, however, has been published on the M serotypes since the near disappearance of pyoderma associated PSGN in developed countries. We were unable to find prior reports of the* emm *44 subtype found in* Patient D*.

Certain factors have been associated with susceptibility to and severity of PSGN. For instance, low birth weights and high BMIs were reported to be associated with increased albuminuria in the Australian aboriginal population which experiences high rates of PSGN [19]. Early life risk factors were not included in our analysis which may be important considerations to identify PSGN patients at risk for complications. A recent case series reported a high rate of AKI in children of Maori or Pacific Islander ancestry with children of European ancestry relatively spared; however GFR was not calculated or BUN presented so direct comparison to our study is not possible [15]. According to the United States 2010 Census, the race/ethnicity demographics of the Columbus, Ohio metropolitan area, is approximately 61.5% non-Hispanic Caucasian, 28% African American, 4% Asian, 5.6% Hispanic, and 0.4% Native American or Pacific Islander. Because the mean GFR ± SD in our series was 45 ± 23 mL/min/1.73 m^2^, our results highlight that children in the United States population may also frequently experience AKI during PSGN. The role of race or ethnicity in PSGN susceptibility remains an area for future investigation.

The PSGN patients in our series who had positive ANA or ANCA titers had particularly severe courses which often included the need for acute hemodialysis. On review of the literature a cluster of severe postinfectious glomerulonephritis cases was reported in Brazil following outbreak caused by group C* Streptococcus zooepidemicus* [20]. The frequency of severe PSGN over time from group A organisms has not been studied to our knowledge; however other populations did report the association between severe PSGN and increased ANCA levels [21].

The study is limited by potential selection bias. We cannot exclude that mild cases of PSGN were cared for by community primary care providers or outside hospitals. This study was also limited by the absence of 24-hour urines to quantify proteinuria in the setting of AKI and our inability to obtain race, ethnicity, gestational age at birth, and birth weights in our medical records.

In the current era, no epidemiologic studies have been published on M types in PSGN. Different strains and virulence factors may be the cause of more severe disease seen in the past. It is not known whether the increased rate of severe PSGN will be isolated to the reported year. We submit that the relevance of the described cohort to PSGN patients includes that PSGN should be considered in cases of severe nephritis even when the presentation is consistent with other types of nephritis. Additionally thrombocytopenia may be associated with PSGN; however it is not clear if the PSGN alone is causative. Testing for thrombotic microangiopathies may be a consideration for future patients with both PSGN and low platelets. Additionally determination of M serotype of organisms resulting in severe PSGN may be helpful in determining if a severe clinical course is based on the infecting organism or underlying patient characteristics.

## Figures and Tables

**Table 1:** Patient demographics.

Patient	Sex	Age (yr)	f/u time (yr)
1	M	6	<1
2	M	7	<1
3	M	8	1
4	F	15	1
5	F	6	<1
6	M	6	<1
7	M	7	0
8	F	8	2
9	M	6	<1
10	M	8	5
11	F	6	1
12	M	12	1
13	M	11	<1
14	F	11	<1
15	F	5	<1
16	M	7	<1
17	M	10	<1

**Table 2:** BP and urine findings during acute PSGN and at follow-up.

Patient	BP (mmHg)		BP%^∧^		Urine heme/RBCs per HPF	
	Initial	f/u	Initial	f/u	Initial	f/u
1	116/76		95	NA	409	79
2	90/60	111/67	50	90	44	Large
3	119/86	107/69	95	95	1011	Trace
4	173/123	126/68	99	90	LRG	Large
5	119/72	107/65	99	90	LRG	28
6	109/74	109/74	90	90	401	Large
7	81/59	89/60	50	25	236	Trace
8	123/66	100/60	99	50	266	Neg
9	138/80	113/62	99	90	776	Large
10	118/73	118/65	95	75	307	Neg
11	114/76	95/60	95	75	1481	Neg
12	141/73	113/63	99	80	573	Neg
13	137/83	128/69	99	96	99	Large
14	131/92	104/67	99	50	247	15
15	149/106	104/70	99	85	LRG	Mod
16	127/53	113/59	99	89	117	Mod
17	136/74	116/65	99	95	23	Small

NA: not available; ^∧^BP% by age, height, and gender.

**Table 3:** Serum lab results during acute PSGN and at follow-up.

Patient	Creatine (mg/dL)		eGFR (mL/min/1.73 m^2^)		BUN		Albumin		C3 (mg/dL)	
	Peak	f/u	Nadir	f/u	Peak	f/u	Peak	f/u	Initial	f/u
1	0.91	0.55	56	93	28	16	3.3	4.6	11	90
2	0.64	0.55	83	96	20	NA	3.8	3.8	42	97
3	7.03	0.56	8	104	120	21	2.6	4.4	29	110
4	1.39	0.87	48	75	36	15	3.2	3.7	17	127
5	0.8	NA	56	NA	18	NA	3.2	NA	15	123
6	0.7	0.45	74	115	22	NA	3.9	NA	20	137
7	0.67	0.48	73	102	26	8	2.6	NA	27	91
8	2.45	0.49	22	115	52	12	2.4	4.2	9	123
9	0.99	0.46	52	110	29	12	3.1	4.7	40	168
10	10.4	0.97	6	69	208	22	2.2	4.1	30	119
11	3.51	0.39	14	136	93	13	3.1	4.1	25	120
12	3.62	0.45	16	130	91	NA	2.5	4	7	123
13	1.64	0.58	40	114	82	13	2.8	4.4	6	139
14	1.8	0.78	36	75	92	14	3.1	NA	15	93
15	0.73	0.45	64	101	26	13	3.1	NA	12	125
16	3.43	0.45	16	119	105	12	NA	NA	11	93
17	1.29	0.48	48	128	46	21	4	4	21	114

NA: not available.

**Table 4:** Patients with atypical PSGN.

Pt	eGFR	BUN initial : peak	K^+^ initial : peak	% fluid overload	BP initial : peak	RRT indication	% crescents	Serology	Atypical findings
3	10	113 : 120	5.6 : 5.6	10.8	119/86 : 145/106	AKI, fluid overload, anuria	66%	ANA +1 : 160	Thrombocytopenia
10	6	208 : 208	6.1 : 6.1	1.9	118/73 : 131/85	AKI, hyperK^+^	85%	ANA +1 : 80	Seizures
11	18	36 : 93	4.7 : 6.6	7.5	114/76 : 131/70	No RRT, hyperK^+^, HTN responded to medical management	30%	cANCA +1 : 20	Mental status changes, neck mass
12	24	64 : 91	4.0 : 5.8	12	141/73 : 159/106	AKI, HTN urgency	None	ANA +1 : 160	Thrombocytopenia

**Table 5:** Comparison of study patients with historical studies in poststreptococcal glomerulonephritis.

Parameter	Study patients	Historical studies	*P* value
BUN > 20 mg/dL	16/17 (94%)	18/45 (40%) [3]	^*∗*^<0.001
		48/138 (35%) [4]	^*∗*^<0.001
		21/35 (60%) [5]	^*∗*^0.01
		36/46 (78%) [6]	0.26

eGFR < 80 mL/min/1.73 m^2^	16/17 (94%)	14/32 (44%) [7]	^*∗*^0.002
		14/29 (48%) [8]	^*∗*^0.004

Hypertensive complications	7/17 (41%)	13/50 (26%) [7]	0.38
		10/35 (28%) [5]	0.55

*∗* refers to statistical significance.
